# Three Cases of Spinocerebellar Ataxia Type 2 (SCA2) and Pediatric Literature Review: Do Not Forget Trinucleotide Repeat Disorders in Childhood-Onset Progressive Ataxia

**DOI:** 10.3390/brainsci15020156

**Published:** 2025-02-04

**Authors:** Jacopo Sartorelli, Maria Grazia Pomponi, Giacomo Garone, Gessica Vasco, Francesca Cumbo, Vito Luigi Colona, Adele D’Amico, Enrico Bertini, Francesco Nicita

**Affiliations:** 1 Unit of Muscular and Neurodegenerative Diseases, Bambino Gesù Children’s Hospital, IRCCS, 00165 Rome, Italy; 2 Medical Genetics Unit, “A. Gemelli” Policlinic University Hospital, IRCCS, 00168 Rome, Italy; 3 Neurology, Epilepsy and Movement Disorder Unit, Bambino Gesù Children’s Hospital, IRCCS, Piazza Sant’Onofrio 4, 00165 Rome, Italy; 4 Department of Neuroscience, Mental Health and Sensory Organs, Faculty of Medicine and Psychology, Sapienza University of Rome, 00185 Rome, Italy; 5 Unit of Neurorehabilitation, Bambino Gesù Children’s Hospital, IRCCS, 00165 Rome, Italy

**Keywords:** spinocerebellar ataxias, movement disorder, pediatric, CAG, myoclonus, SCA

## Abstract

**Background**: Childhood-onset progressive ataxias are rare neurodegenerative disorders characterized by cerebellar signs, sometimes associated with other neurological or extra-neurological features. The autosomal dominant forms, known as spinocerebellar ataxias (SCAs), linked to trinucleotide (i.e., CAG) repeat disorders, are ultra-rare in children. We describe three patients from two unrelated families affected by spinocerebellar ataxia type 2 (SCA2) and present a literature review of pediatric cases. **Methods**: The patients’ clinical and genetic data were collected retrospectively. **Results**: The first case was a 9.5-year-old boy, affected by ataxia with oculomotor apraxia and cerebellar atrophy, subcortical myoclonus, and peripheral axonal sensitive polyneuropathy caused by a pathologic expansion in *ATXN2*, inherited from his asymptomatic father. Two brothers with familial SCA2 presented neurodegeneration leading to early death in one case and progressive ataxia, parkinsonism, and epilepsy with preserved ambulation at age 18 years in the second. To date, 19 pediatric patients affected by SCA2 have been reported, 3 of whom had a phenotype consistent with progressive ataxia with shorter CAG repeats, while 16 had more severe early-onset encephalopathy, with longer alleles. **Conclusions**: Although they are ultra-rare, trinucleotide repeat disorders must be considered in differential diagnosis of hereditary progressive ataxias in children, especially considering that they require targeted genetic testing and can manifest even before a parental carrier becomes symptomatic. Thus, they must also be taken into account with negative family history and when Next-Generation Sequencing (NGS) results are inconclusive. Notably, the association between cerebellar ataxia and other movement disorders should raise suspicion of SCA2 among differential diagnoses.

## 1. Introduction

Cerebellar ataxia is a disorder of balance and coordination often characterized by dysmetria, intention tremor, dysarthria, and ocular movement abnormalities [1]. It can be due to both acquired and hereditary (i.e., genetic) causes. Acquired ataxias can be linked to a variety of etiologies, such as infections, autoimmune or paraneoplastic disorders, neoplasms, toxic exposure, nutritional deficits, and endocrine diseases. Different hereditary conditions can present with cerebellar signs as part of the disease (i.e., leukodystrophies, mitochondrial diseases, multisystem atrophy, exc.) [2]. In addition to these, genetic cerebellar ataxias are rare disorders that can ideally be subdivided into two forms: congenital ataxias present with early-onset (e.g., during the first few months of age) hypotonia, developmental delay, and oculomotor abnormalities [3], while progressive ataxias have a later (e.g., from childhood to adulthood) onset of worsening cerebellar signs. From a genetic point of view, both autosomal dominant, autosomal recessive, and X-liked inheritance is encountered [1,3]. Autosomal dominant ataxias are known as spinocerebellar ataxias (SCAs) and can be caused by both gene variants and trinucleotide (i.e., CAG) repeat expansion. This mechanism is linked to the majority of cerebellar ataxias in adulthood, with an overall prevalence in Europe between 1 and 3 every 100.000 individuals. The most frequent forms are SCA3, SCA2, SCA1, SCA7, SCA6, SCA17, and dentatorubro-pallidoluysian atrophy (DRPLA) [4,5]. These disorders become manifest when the allele reaches a specific size for each gene, with a possible increase through generations due to allele instability, especially during paternal transmission. A bigger size is linked with a progressive reduction in the age at symptom onset, named the anticipation phenomenon, leading to pediatric-onset forms in ultra-rare cases [1,6,7].

Expansions over 34 CAG repeats in *ATXN2* gene are linked to adult-onset SCA2, a severe neurodegenerative condition characterized by progressive cerebellar ataxia, saccadic slowing, extrapyramidal movement disorder (e.g., parkinsonism and myoclonus), and subsequent brainstem involvement [8]. Pediatric SCA2 cases are almost anecdotal [9,10,11,12,13,14,15,16,17,18,19]. In this article, we aim to emphasize the importance of considering CAG repeat disorders in the differential diagnosis of childhood-onset cerebellar ataxia, describing three patients from two unrelated families affected by SCA2 and a literature review of pediatric cases, trying to define differences in this condition across ages and potential genotype–phenotype correlations and suggesting possible diagnostic clues.

## 2. Materials and Methods

The study participants were under follow-up at the Muscular and Neurodegenerative diseases Unit or at the Neurorehabilitation Unit of Bambino Gesù Children’s Hospital in Rome. Clinical and laboratory data were collected retrospectively. The parents of the participants gave written informed consent for genetic testing and for participation in research studies.

Genetic testing was variably performed in the reported subjects. Trio NGS was performed using the KAPA HyperExome Probes (Roche, Basel, Switzerland) kit on the NovaSeq6000 platform (Illumina, San Diego, CA, USA), reaching 380× medium coverage. Only regions with a minimum of 30× coverage were included in the analysis, with an estimated sensitivity and specificity higher than 99%. A bioinformatic analysis was performed through the BWA Aligner/DRAGEN Germline and Pipeline/DRAGEN Enrichment system. Sequences were aligned with the GRCh37 human reference genome, and variants were filtered and prioritized using Geneyx Analysis software Version 5.15 (a knowledge-driven NGS analysis tool powered by the GeneCards Suite). Variants were classified according to the ACMG criteria. The gene selection was made according to the HPO, OMIM, and GeneReviews databases. A CAG expansion analysis of certain SCA-related genes (*ATXN1, ATXN2, ATXN3, CACNA1A, ATXN7, PPP2R2B*, and *TBP*) was completed using fluorescent primer PCR (with primer sequences provided in Supplementary Table S1), capillary electrophoresis on an ABI PRISM 35000 Genetic Analyzer (Applied Byosystems, Waltham, MA, USA), and a data analysis using GeneMapper 4.1 (Applied Byosystems).

Literature review was performed on PubMed database (https://pubmed.ncbi.nlm.nih.gov/) (last search: 30 November 2024) by searching for a combination of the following terms: “spinocerebellar ataxia type 2”, “pediatric”, “children”, “*ATXN2*”, and “ataxin-2”. The references of the selected articles were also screened for additional cases.

## 3. Results

### 3.1. Case Reports

**Figure 1:**
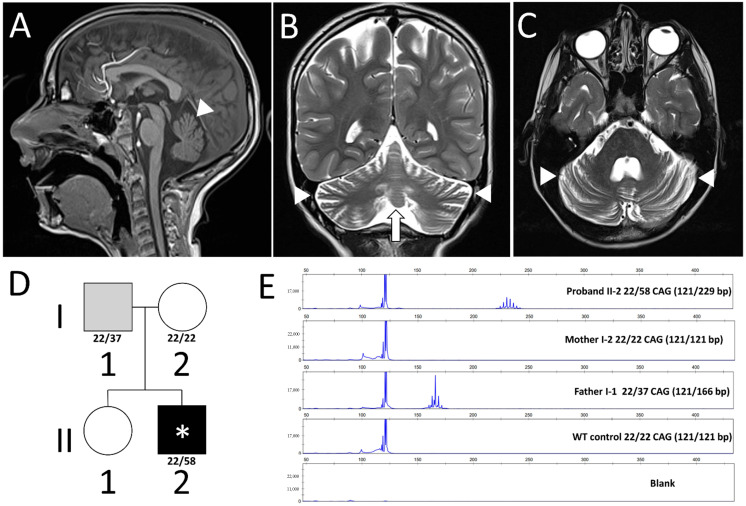
Brain MRI, genetic data, and pedigree of subject 1. (**A**–**C**) T1 sagittal, T2 coronal, and T2 axial representative images showing cerebellar cortical ((**A**–**C**) arrowheads) and vermian ((**B**) arrow) atrophy in subject 1. (**D**) Subject 1’s family tree showing the father as the asymptomatic carrier (I-1, gray), and the affected subject 1 (II-2, black with an asterisk). CAG repeats of *ATXN2* alleles were reported when available. Squares are used for male and circles for female subjects. Black indicates affected individuals and gray asymptomatic subjects, with asterisks denoting pediatric cases. (**E**) Capillary electrophoresis images showing the allele dimensions within subject 1’s family: the affected subject carries a 58 CAG allele (229-base-pair PCR product), expanded from a paternal 37 CAG allele (166 bp PCR product). The healthy mother (I-2) presents 22 CAG alleles as the wild-type control. The asymptomatic older sister (II-1) did not undergo genetic testing.

Subject 1 (II-2, Figure 1D) is a 9.5-year-old boy, the second child of non-consanguineous parents with no family history of neurologic conditions. Pregnancy was uneventful. He was born at term through a caesarean section due to breech presentation. He had a normal neonatal period and regular postnatal growth, with a weight and height at the lower centiles, as well as regular psychomotor development (independent walking at 15 months, adequate language). He was described as clumsier than his peers during ambulation or running, with difficulties in motor coordination. At the age of 8 years, he was referred for a neuropsychological evaluation due to difficulties in his school performance, and he was diagnosed with a learning disability, with poor reading and comprehension, dysgraphia, and dyscalculia. After 3 months, he was sent to our emergency department due to an acute asthmatic attack, and during medical evaluation, signs of cerebellar ataxia were evident. He underwent a computed tomography scan, evidencing cerebellar atrophy, and he was then admitted for further testing. The neurologic examination showed good interaction with intelligible dysarthria. Truncal and appendicular ataxia was apparent: he could sit and stand with his feet together but in both cases with body oscillations. He was not able to stand in tandem position. Independent walking was possible with an ataxic, broad-based gait, difficulties in turning, forward truncal flection, and a reduced arm swing. Distal myoclonus mainly involving the hands was also evident. Muscular tone was normal, with normal deep tendon reflexes and down-going plantar responses. Oculomotion was characterized by difficulties in starting and completing eye movements, with oculomotor apraxia and possible up-gaze paralysis, without nystagmus. A comprehensive neuropsychological evaluation revealed a mixed learning disability, with borderline intellectual functioning (Wechsler Intelligence Scale for Children, WISC-IV, total IQ: 77; main issues in perceptual reasoning, working memory, and processing speed). Brain magnetic resonance imaging was then performed (Figure 1A–C), confirming cerebellar atrophy with enlargement of the infratentorial subarachnoid space. The extended neurophysiologic study (including an electroencephalogram, an electroretinogram, multimodal-evoked potential, and nerve conduction studies (NCSs)) was unremarkable. The ophthalmologic and cardiologic evaluations were both normal, as well as a broad ataxia-related blood examination panel (vitamin E, copper, caeruloplasmin, albumin, cholesterol, immunoglobulin subclasses, alpha-fetoprotein, cholestanol, very long-chain fatty acids, and phytanic and pristanic acid). Of note, his neurofilament light chain levels were 318 pg/mL (>95th centile for age). Genetic testing was then performed, and trio-based whole-exome sequencing did not detect pathogenic variants. Finally, a CAG expansion analysis of more frequent SCA-related genes (*ATXN1, ATXN2, ATXN3, CACNA1A, ATXN7, PPP2R2B,* and *TBP*) allowed for the identification of a pathologic 58-triplet expansion in *ATXN2* (Figure 1E, subject II-2, top line). After obtaining consent, a segregation analysis was performed in the healthy parents, revealing a 37-triplet expansion in the 44-year-old father (Figure 1E, subject I-1, third line). During the 1-year follow-up, subject 1 showed an increment in his movement disorder that was better characterized through a polygraphic EEG, evidencing subcortical myoclonus at rest and during movement, associated with action tremor. Ataxia remained stable, resulting in a 17/40 score on the Scale for the Assessment and Rating of Ataxia (SARA) [4]. Repetition of the NCSs evidenced the initial signs of axonal sensitive polyneuropathy. 

**Figure 2:**
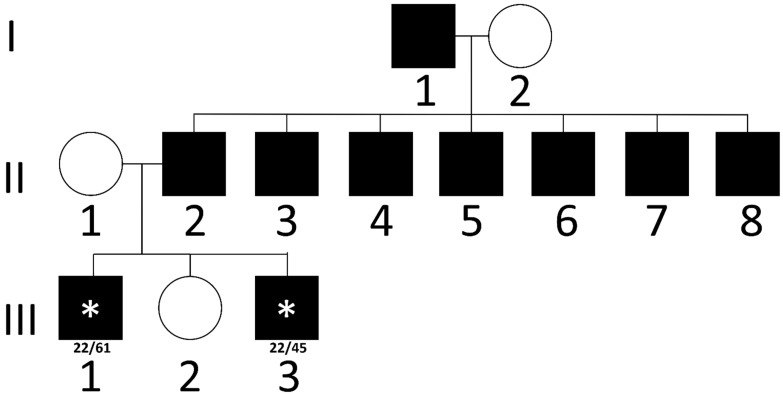
Family tree of Subjects 2 and 3. Subjects 2 (III-1, black with asterisk) and 3 (III-3, black with asterisk) family tree showing the affected father (II-2, black), uncles (II-3 to 8, black) and grandfather (I-1, black). CAG repeats of *ATXN2* alleles have been reported when available. Squares are used for male, circles for female subjects. Black indicates affected individuals, with asterisk in pediatric cases.

Subject 2 (III-1, Figure 2) had initial normal development and was first referred at age 6 years due to limb tremor. He was the first son of non-consanguineous parents. His father died at 39 years of age after being diagnosed with SCA2, and six paternal uncles were affected by the same disease. After a clinical evaluation that evidenced ataxic signs, a brain MRI was performed, showing cerebellar atrophy, and genetic testing confirmed the presence of a pathologic 61 CAG *ATXN2* allele. The disease progressed in subsequent years, with ambulation loss at 10 years of age and worsening feeding abilities up to dysphagia that required PEG tube placement at 11 years. Due to acute respiratory insufficiency after an airway infection, at 12 years, a tracheostomy was placed, and invasive ventilation was performed at bedtime. At 17 years, he underwent different surgical procedures: adductor tenotomy for his lower limb contractures, Nissen fundoplication for gastroesophageal reflux disease, and salivary duct ligation for drooling. A last evaluation was performed at the age of 17 years 4 months evidencing apostural tetraplegia with dyskinesia and limb myoclonus. Severe scoliosis, diffuse muscle hypotrophy, and diffuse limb deformities due to tendon contractures were also reported. He died at the age of 18 years due to respiratory infectious disease.

Subject 3 (III-3, Figure 2) is a 20-year-old man, the younger brother of patient 2, and the third son in the family (a healthy sister has also been reported). He presented with febrile seizures from the age of 6 months to 4 years and mild developmental delay, with learning difficulties. At the age of 17 years, he had generalized tonic–clonic seizures, and an electroencephalogram (EEG) showed focal abnormalities and photosensitivity. A brain MRI evidenced cerebellar atrophy. Levetiracetam therapy was started, but complete seizure control was reached after the introduction of Valproate as an add-on. Genetic testing was then performed, confirming the presence of a 45 CAG allele in *ATXN2*. The last evaluation was performed at the age of 18 years, showing bradykinesia, limb and truncal ataxia, and an eye movement disorder with slow and hypermetric saccades. 

### 3.2. The Literature Review

To date, 19 pediatric (i.e., 0–18-year-old) cases affected by SCA2 have been reported [9,10,11,12,13,14,15,16,17,18,19], with the number of CAG repeats ranging from 62 to 884 (Table 1). Paternal inheritance was more frequent, in 14 out of 19 of cases, while an affected mother was present in 4 cases. In one case, the allele transmission was not reported. The parental alleles ranged from 39 to 51 CAG repeats. The age at symptom onset was neonatal (i.e., 0–27 days of life) in 4 out of 19 of the subjects, infantile (i.e., 28 days–1 year of life) in 13 out of 19, and during childhood (i.e., 1–12 years) in 2 out of 19.

The majority of subjects (16 out of 19) harbored larger alleles (92–884), and they presented with early-onset encephalopathy characterized by hypotonia (11 out of 16), severe developmental delay (11 out of 16), and possible loss of the few acquired milestones (3 out of 16). These signs were variably accompanied by seizures (6 out of 16; i.e., tonic, myoclonic seizures or infantile spasms) and/or EEG abnormalities (9 out of 16; hypsarrhythmia in 6 cases), eye movement alterations (i.e., nystagmus, strabismus, erratic eye movements), extrapyramidal movement disorder (3 out of 16; i.e., chorea, myoclonus, dystonia), spasticity (2 out of 16; i.e., paraplegia and tetraplegia), or neuropathy (1 out of 16). Brain MRI often showed cerebellar atrophy (13 out of 16), associated in some cases with supratentorial (6 out 16) and/or brainstem atrophy (3 out of 16) and T2 white matter hyperintensity (5 out of 16). Neonatal forms (3 out of 16) presented with severe hypotonia with possible apneic episodes (2 out of 3) or limb spasticity and dysphagia (1 out of 3). The main forms of extra-neurologic involvement included pigmentary retinopathy (12 out of 16) and dysphagia (7 out of 16).

A minority of pediatric SCA2 cases (3 out of 19, italics in Table 1) presented with fewer repeats (62–75) and a phenotype characterized by neurodevelopmental disorder (3 out of 3) and subsequent neurodegeneration featuring altered saccades (3 out of 3) and progressive ataxia, leading to a loss of acquired milestones (3 out of 3). Nystagmus was the first sign at the age of 2 months in one case, while a second subject presented with an extrapyramidal movement disorder (i.e., choreoathetosis, non-epileptic myoclonus, and dystonia), peripheral neuropathy, and a behavioral disorder (i.e., polyphagia with obesity). Brain MRI was performed in one individual, showing cerebro-cerebellar and brainstem atrophy. Two patients presented with dysphagia and drooling and subsequently developed sphincter incontinence.

**Figure 3:**
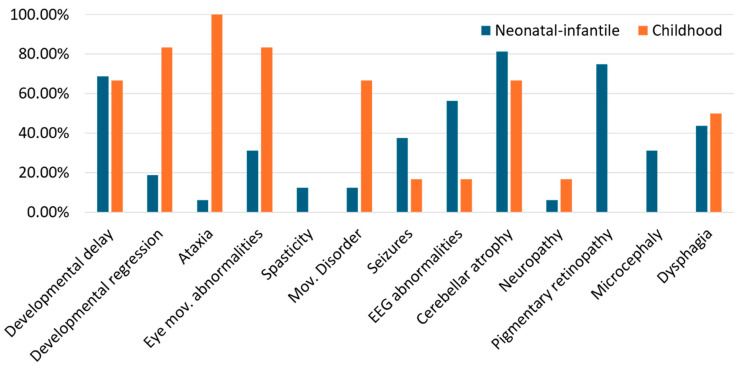
Summary of clinical features of patients with neonatal/infantile- vs. childhood-onset SCA2. Percentage of reported subjects with neonatal/infantile- (*n* = 16) vs. childhood-onset (*n* = 6) SCA2, presenting the main clinical characteristics. Mov. = movement.

The clinical features of the patients (also considering those reported in our work) with the infantile- vs. childhood-onset forms are summarized in Figure 3.

## 4. Discussion

SCA2 in adulthood is characterized by a combination of gait ataxia, dysarthria, and action tremor, often with parkinsonian signs (i.e., rigidity and bradykinesia). Other typical manifestations are early and marked saccadic slowing, possible initial hyperreflexia, followed by hyporeflexia and myoclonus. Disease progression leads to ambulation loss, dysphagia, and incontinence. Atrophy of the cerebellum, the middle cerebellar peduncle, and the midbrain with subsequent cortical frontotemporal involvement is almost always present [8]. A significant inverse correlation has been established between age at onset and CAG repeat length in SCA2, with a mean age at onset ranging from 60 years for people with 34–35 CAG alleles to 23 years for those with 44–45 CAG alleles and then to under 20 years for those with larger alleles [20]. 

At pediatric age, SCA2 can manifest from neonatal period to childhood. Neonatal and infantile forms are more frequent and have an overlapping encephalopathic phenotype, characterized by developmental delay (with poor or no milestone acquisition), hypotonia, seizures, and pigmentary retinopathy [9,10,11,12,13,14,15,16]. Childhood-onset neurodegeneration has been described in only three individuals to date [17,18,19], and in our article, we report on three additional cases. This second childhood-onset presentation is characterized by a possible neurodevelopmental disorder at the onset of the disease, followed by progressive ataxia, regression, eye movement abnormalities, and, over time, the appearance of subcortical myoclonus and alterations in brainstem functions, with dysphagia and drooling. Notably, the six SCA2 cases harbor smaller CAG expansions (e.g., medium-size pathologic alleles) compared to those of other affected pediatric subjects, and extrapyramidal movement disorders (i.e., myoclonus, choreoathetosis, dystonia, and/or parkinsonism) are encountered in most cases (4 out of 6), thus suggesting a potential diagnostic clue. From the literature reports, neuroimaging findings show a typical progression of neurodegeneration, characterized by initial cerebellar and early brainstem involvement, with subsequent supratentorial atrophy and secondary white matter alterations [9,10,11,12,13,14,15,16,17,18,19]. Few longitudinal data are available in the childhood-onset SCA2 subgroup, and in our report, we present two brothers under follow-up until early adulthood. The older, carrying a 61 CAG allele, presented with severe neurodegeneration leading to tetraplegia with PEG tube placement and ventilation support after 5 to 6 years from disease onset, while the younger, carrying a 45 CAG allele, had progressive ataxia with parkinsonism and a preserved (ataxic) gait at the age of 18 years.

Ataxin-2 is a basic protein composed of two globular domains. It appears to be located within the Golgi and endoplasmic reticulum and to be involved in endocytosis, mTOR signal modulation, ribosomal translation modification, and mitochondrial function. However, its role in physiology and disease still needs to be fully understood. This protein is highly expressed by Purkinje cells, and its levels appear to increase over time. The ataxin-2 N-terminal region contains a polyglutamine tract coded by a CAG repeat motif, whose expansion leads to neurodegeneration through various possible mechanisms, including altered autophagy, the formation of aggregates, and neuroinflammation [21]. Healthy individuals carry a range of 14–31 residues (29–31 residues have been associated with an increased risk of amyotrophic lateral sclerosis), while alleles with 32 or more residues have been linked with SCA2. The number of repeats has been shown to expand in families over successive generations, resulting in an earlier onset and faster progression, but with possible clinical heterogeneity within the same family [20]. A potential correlation between the number of CAG repeats and disease severity also appears at pediatric age, with medium-size (i.e., 62–75) alleles linked to progressive ataxia, while major repeats (i.e., 92–884) are linked to early-onset encephalopathy. However, there are too few cases to define a clear cut-off, and even within the same phenotypic group, it is not possible to predict the age at onset (e.g., neonatal vs. infantile forms).

Due to the heterogeneity, the GC content, and elongated sequences of the tandem repeat loci, polymerase chain reaction (PCR)-based techniques are required, as short-read NGS is not able to accurately identify the allele sizes [22]. This underscores the importance of considering CAG triplet repeat expansion disorders in the differential diagnosis of childhood progressive ataxias, which are usually approached using NGS as the standard first-line genetic testing. For this purpose, recently, the European Reference Network for Rare Neurologic Diseases (ERN-RND) proposed a diagnostic flowchart for early-onset ataxia [23]. As shown in the subjects included in our report, repeat disorder testing should be the first choice in presence of autosomal dominant family history, especially with the anticipation of signs and symptoms, and must be also considered when NGS testing results are inconclusive for sporadic or apparently isolated cases. Finally, long-read sequencing techniques have also shown promising results in triplet expansion diseases [20]. In the future, their development and diffusion could overcome the limits of currently used genetic tests, possibly changing our diagnostic approach to these genetically heterogeneous disorders.

## 5. Conclusions

Childhood progressive ataxias are genetically heterogeneous diseases that are very rarely caused by a triplet expansion mechanism. Since trinucleotide expansion disorders need targeted testing to be identified, they must be taken into account in the presence of pediatric-onset neurodegenerative ataxia, especially with an affected parent, but also in sporadic or apparently isolated cases when the NGS results are inconclusive, before the routine application of long-read WGS sequencing techniques. Clinically, the distinctive features of pediatric SCA2 appear to be early saccadic abnormalities and extrapyramidal movement disorders, coupled with childhood-onset ataxia being caused by medium-size expansions, while severe infantile encephalopathy with developmental delay, seizures, and pigmentary retinopathy is caused by longer alleles.

## Figures and Tables

**Table 1:** Pediatric SCA2 patients from the available medical literature. List of abbreviations: n/a, not available; y, yes; mov., Movement. Childhood-onset neurodegenerative forms have been highlighted in italics.

Patient	1	2	3	4	5	6	7	8	9	10	11	12	13	14	15	16	*17*	*18*	*19*	*Subject 1*	*Subject 2*	***Subject 3***
**Repeat number**	884	104	320	124	92	300	750	500	>200	220	200	500	350	400	230	220	*69–75*	*70*	*62*	*58*	*61*	*45*
**Inheritance (allele)**	Maternal (49)	Paternal (n/a)	Paternal (47)	Paternal (45)	Paternal (51)	Paternal (43)	Paternal (40)	Paternal (40)	Maternal (45)	Maternal (43)	Paternal (42)	Maternal (45)	Paternal (40)	Paternal (43)	Paternal (40)	Paternal (43)	*Paternal (39)*	*Paternal (40)*	*n/a*	*Paternal (37)*	*Paternal (n/a)*	*Paternal (n/a)*
**Age of onset**	neonatal	neonatal	6 months	6 months	4 months	2 months	infantile	3 months	3 months	2 weeks	6 months	10 months	3 months	10 months	11 months	neonatal	*2 years*	*5 years*	*2 months*	*8 years*	*6 years*	*6 months*
**Signs or symptoms at onset**	Nystagmus, dysphagia	Hypotonia, dysphagia	Hypotonia, developmental regression	Hypotonia, eye movement abnormalities, myoclonus	Developmental delay, hypotonia, dyskinesia	Hypotonia	Developmental delay, microcephaly	Developmental regression	Focal seizures	Apnea episodes	Hypotonia	Hypotonia, developmental delay	Hypotonia, seizures	Hypotonia, developmental delay	Hypotonia, developmental delay	Hypotonia, apnoea episodes	*Ataxia, progressive movement disorder*	*Ataxia, developmental delay*	*Eye movement abnormalities*	*Learning disability, cerebellar ataxia*	*Limb tremor*	*Febrile seizures, mild developmental delay*
**Developmental delay**	y		y	y	y		y			y	y	y	y	y	y		*y*	*y*	*y*			*y*
**Developmental regression**						y	y	y									*y*	*y*	*y*		*y*	*y*
**Ataxia**	y																*y*	*y*	*y*	*y*	*y*	*y*
**Eye movement abnormalities**	y			y	y							y			y		*y*	*y*	*y*	*y*		*y*
**Spasticity**	y												y									
**Mov. Disorder**		y		y													*y*			*y*	*y*	*y*
**Seizures**		y				y		y	y	y	y											*y*
**EEG abnormalities**				y		y		y	y	y	y	y	y		y							*y*
**Cerebellar atrophy (MRI)**	y	y	y	y	y	y	y	y	y		y	y	y		y				*y*	*y*	*y*	*y*
**Neuropathy**					y												*y*			*y*		
**Pigmentary retinitis**		y	y	y	y	y	y	y		y	y	y		y		y						
**Microcephaly**					y			y	y	y	y											
**Dysphagia**	y	y			y		y		y					y		y		*y*	*y*		*y*	
**Comorbidities**					Dysmorphic features, PEG placement 1 year, GERD		Autonomic disfunction, optic nerve atrophy		Autonomic instability		Optic nerve atrophy		Visual impairment				*Vasomotor instability, polyphagy, obesity*	*Incontinence, drooling*	*Incontinence, drooling*	*Borderline intellectual functioning*	*Lower limb contractures, scoliosis, respiratory insufficiency with tracheostomy, death at 18 years*	
**Reference**	[9]	[10]	[11]	[12]	[13]	[14]	[14]	[14]	[14]	[14]	[14]	[15]	[15]	[15]	[15]	[16]	[17]	[18]	[19]	*This work*	*This work*	*This work*

## Data Availability

The original contributions presented in this study are included in the article; further inquiries can be directed to the corresponding author.

## Supplementary Materials

The following supporting information can be downloaded at: https://www.mdpi.com/article/10.3390/brainsci15020156/s1, Table S1: Sequences of fluorescent PCR primers used for CAG expansion analysis of some SCA-related genes (*ATXN1*, *ATXN2*, *ATXN3*, *CACNA1A*, *ATXN7*, *PPP2R2B*, *TBP*).
