# Supplementary material for: Three Cases of Spinocerebellar Ataxia Type 2 (SCA2) and Pediatric Literature Review: Do Not Forget Trinucleotide Repeat Disorders in Childhood-Onset Progressive Ataxia
Source: Brain Sci. 2025 Feb 4;15(2):156. doi: 10.3390/brainsci15020156 (PMC11853045; doi:10.3390/brainsci15020156)
Supplement: Supplementary file 1 [file brainsci-15-00156-s001.zip › brainsci-3431542-supplementary.pdf]

Supplementary Table S1

Sequences of fluorescent PCR primers used for CAG expansion analysis of some SCA-related genes (*ATXN1, ATXN2, ATXN3, CACNA1A, ATXN7, PPP2R2B, TBP*).

**Primer**	**Sequence**
ATXN1-F	5' NED-CCAACATGGGCAGTCTGAG
ATXN1-R	AACTGGAAATGTGGACGTAC
ATXN2-F	5' FAM-GGGCCCCTCACCATGTCG
ATXN2-R	CGGGCTTGCGGACATTGG
ATXN3-F	5' VIC-CCAGTGACTACTTTGATTCG
ATXN3-R	TGGCCTTTCACATGGATGTGAA
CACNA1A-F	5' FAM-CACGTGTCCTATTCCCCTGTGATCC
CACNA1A-R	TGGGTACCTCCGAGGGCCGCTGGTGG
ATXN7-F	5' VIC-TGTTACATTGTAGGAGCGGAA
ATXN7-R	CACGACTGTCCCAGCATCACTT
PPP2R2B-F	5' FAM-TGCTGGGAAAGAGTCGTG
PPP2R2B-R	GCCAGCGCACTCACCCTC
TBP-F	5' VIC-CCCTATCTTTAGTCCAATGATGCCT
TBP-R	CTGTGAGTGGAAGAGCTGTGGT
